# Effects of electrostatic therapy on nighttime sleep and daytime symptoms in patients with chronic insomnia: Evidences from an open label study

**DOI:** 10.3389/fnins.2022.1047240

**Published:** 2023-01-06

**Authors:** Yanyuan Dai, Qingsong Qin, Baixin Chen, Le Chen, Qimeng Sun, Alexandros N. Vgontzas, Maria Basta, Yun Li

**Affiliations:** ^1^Department of Sleep Medicine, Shantou University Mental Health Center, Shantou University Medical College, Shantou, Guangdong, China; ^2^Sleep Medicine Center, Shantou University Medical College, Shantou, Guangdong, China; ^3^Department of Psychiatry, Sleep Research and Treatment Center, College of Medicine, The Pennsylvania State University, Hershey, PA, United States; ^4^Department of Psychiatry, University Hospital of Heraklion, Heraklion, Greece

**Keywords:** insomnia, insomnia with short sleep duration, transcranial electric stimulation, electrostatic therapy, treatment

## Abstract

**Introduction:**

Transcranial electric stimulation (TES) is a neuromodulation approach that applies low-intensity electrical current to the brain and has been proposed as a treatment for insomnia. Electrostatic therapy is a kind of TES and people do not have a feeling of electrical stimuli when the voltage of static electricity is lower than 2,000 volts. However, no studies have examined the effects of electrostatic therapy on objective sleep and daytime symptoms in patients with insomnia.

**Materials and methods:**

Thirty chronic insomnia patients were included. All patients received a 6 week electrostatic therapy and three comprehensive assessments including two consecutive polysomnography (PSG) and daytime symptoms assessments, at pre-treatment, 3 week and 6 week of treatment. Insomnia Severity Index (ISI) was used to assess the severity of insomnia. Multiple sleep latency test (MSLT), Epworth Sleepiness Scale (ESS), and Flinders Fatigue Scale (FFS) were used to assess objective and self-reported daytime sleepiness and fatigue, respectively. Attention network test (ANT) was used to assess attention levels.

**Results:**

Total ISI scores decreased significantly at 3 weeks (*p* < 0.001) and 6 weeks (*p* < 0.001) after initiation of treatment. Furthermore, objective total sleep time (TST, *p* = 0.020) and sleep efficiency (SE, *p* = 0.009) increased and wake time after sleep onset (*p* = 0.012) decreased significantly after 6 weeks electrostatic therapy. Regarding daytime symptoms, ESS and FFS scores decreased significantly at 3 weeks (ESS, *p* = 0.047; FFS, *p* = 0.017) and 6 weeks (ESS, *p* = 0.008; FFS, *p* = 0.003) after initiation of treatment. Moreover, executive control improved significantly from pre-treatment to 3 weeks (*p* = 0.006) and 6 weeks (*p* = 0.013) and altering network improved significantly at 6 weeks (*p* = 0.003) after initiation of treatment. Secondary analyses showed that TST and SE improved significantly after electrostatic therapy in insomnia patients who slept < 390 min (all *p*-value < 0.05). However, no significant changes regarding TST and SE were observed in insomnia patients who slept ≥ 390 min.

**Conclusion:**

Electrostatic therapy improves both nighttime sleep and daytime symptoms in patients with chronic insomnia. The effect on objective sleep appears to be stronger in patient with objective short sleep duration. Electrostatic therapy might be a therapeutic choice for insomnia patients with difficulty maintaining sleep and not responding to behavioral treatments.

**Clinical trial registration:**

[www.clinicaltrials.gov], identifier [ChiCTR2100051590].

## 1. Introduction

Insomnia is the most prevalent sleep disorder and the second common mental disorder in general clinical practice, with 19–50% of adults reporting symptoms of insomnia in epidemiological studies, with half of the patients suffering from chronic insomnia (Wittchen et al., 2011; Winkelman, 2015; Sutton, 2021). Insomnia does not only manifest as nighttime symptoms (i.e., difficulty falling or staying asleep, waking too early), but also accompanied by daytime functional impairment (i.e., fatigue, daytime sleepiness, and attention impairment) (Sutton, 2021). Previous studies have shown that insomnia is associated with increased risk of psychiatric (i.e., depression and anxiety) (Riemann, 2007; Staner, 2010; Li et al., 2016; Chellappa and Aeschbach, 2022) and cardiometabolic morbidity (Vgontzas et al., 2009a,b; Li et al., 2015; Bertisch et al., 2018; Laaboub et al., 2022), as well as an economic burden on the overall healthcare system (Taddei-Allen, 2020; Streatfeild et al., 2021). Cognitive behavioral treatment -insomnia (CBT-I) is considered “first line” treatment, however there is a large number of patients that do not respond well to this treatment modality (Bathgate et al., 2017) and may respond better to biological treatments (Vgontzas et al., 2020; Li et al., 2021).

Neuromodulation technique, such as transcranial direct current stimulation (tDCS), transcranial alternating current stimulation (tACS), and cranial electrical stimulation (CES) have been suggested as potentially useful for insomnia. And CES has been approved by the US Food and Drug Administration for the treatment of insomnia, anxiety, and depression (Shekelle et al., 2018). Limited prior studies have mainly focused on direct and alternating current stimulation on insomnia and the findings were inconsistent. A randomized double blind controlled trial suggested that tACS improved self-reported insomnia severity in patients with insomnia (Wang et al., 2020). Moreover, a study involving 57 participants showed no significant changes in total sleep time (TST) following a 5 day CES treatment (Lande and Gragnani, 2013), while another study involving 19 psychiatric patients showed significant reduction of global insomnia ratings with by a 14 day CES treatment (Feighner et al., 1973). Another study involving 10 participants showed significant reduction of sleep latency measured with electroencephalography (Weiss, 1973). Furthermore, insomnia is a disorder affecting not only nighttime sleep, but also daytime function (i.e., fatigue, daytime sleepiness, mood status, and attention impairment). However, none of these studies has examined the effect of transcranial electric stimulation (TES) on daytime insomnia symptoms that interfere with functioning and mood of the chronic insomnia patients. Electrostatic therapy is a new kind of TES that generates electrostatic potentials by applying electrostatic patches to the skin. To date, only one study reported electrostatic therapy improved self-reported sleep quality in patients with insomnia (Wang et al., 2019). However, no study has examined the effects of electrostatic therapy on objective sleep and daytime function in insomnia patients. In this study, we aimed to evaluate the efficacy of the electrostatic therapy on subjective and objective nighttime sleep and daytime symptoms (i.e., fatigue, daytime sleepiness, mood status, and attention levels) of patients with chronic insomnia, and provide clinical insights for the treatment of insomnia.

## 2. Materials and methods

This was a single arm open-label pre- and post-intervention design study with a 6 week electrostatic therapy intervention. This study was approved by the Research Ethics Board of Shantou University Mental Health Center and informed consent was obtained from each participant.

### 2.1. Participants

**FIGURE 1:**
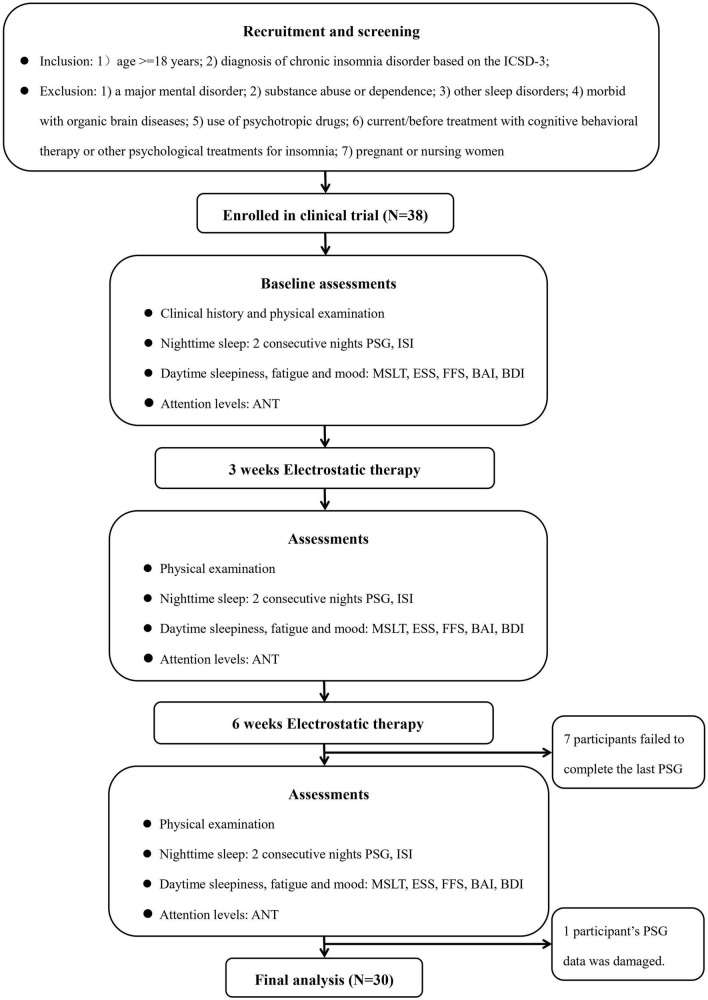
Flow chart. ANT, attention network test; BAI, Beck Anxiety Inventory; BDI, Beck Depression Inventory; ESS, Epworth Sleepiness Scale; FFS, Flinders Fatigue Scale; ICSD-3, the International Classification of Sleep Disorder, third edition criteria; ISI, Insomnia Severity Index; MSLT, multiple sleep latency test; PSG, polysomnography; PSQI, Pittsburgh Sleep Quality Index.

Figure 1 depicts the flow of this study. All the eligible participants were screened according to research protocols by the Sleep Medicine Center of Shantou University Medical College. Inclusion criteria included (1) age ≥ 18 years and (2) diagnosis of chronic insomnia disorder based on the International Classification of Sleep Disorders third edition (ICSD-3) (American Academy of Sleep, 2014). Exclusion criteria included (1) a major mental disorder (e.g., schizophrenia, major depression, and generalized anxiety disorder); (2) substance abuse or dependence; (3) other sleep disorders [e.g., obstructive sleep apnea (OSA), restless legs syndrome, periodic limb movement disorder, and circadian rhythm disorders] based on an overnight polysomnography (PSG) examination, clinical symptoms and/or relevant screening questionnaires; (4) morbid with organic brain diseases (e.g., history of brain trauma, epilepsy); (5) use of psychotropic drugs (e.g., hypnotics, sedative antipsychotics or sedative antidepressants) for insomnia currently or in the past 1 month; (6) current/past treatment with cognitive behavioral therapy or other psychological treatments for insomnia; or (7) pregnant or nursing women. In this study, the advertisements were distributed through social media (i.e., WeChat), radio, newspapers, and we also recruited participants on-site in communities and colleges. In the current study, an apnea hypopnea index ≥ 5 events/h was used to define the presence of OSA and a periodic limb movement index ≥ 15 events/h was used to define the presence of periodic limb movement disorder. Initially, a total of 38 adult patients with chronic insomnia were recruited between June 2019 and November 2020. However, three patients dropped off from the study at their last visit due to personal reasons and four patients because of Corona Virus Disease-2019 (COVID-19) pandemic-related traffic restrictions. In addition, one patient’s PSG data was damaged and could not be evaluated. Ultimately, 30 chronic insomnia patients were included in the analyses. Results from power calculation with the G*Power 3.1.9.2 program (Teodorovici, 2022) showed that there was 83.05%, 79.26%, and 90.84% power to reject the null hypothesis and detect a significant time effect of TST, sleep efficiency (SE), and wake time after sleep onset (WASO), respectively in the current study.

### 2.2. Procedures

Electrostatic therapy is a new kind of TES and generates electrostatic potentials by applying electrostatic patches to the skin. All patients included received electrostatic therapy (absolute value 1,500 ± 3% V) during their nighttime sleep by applying two 20 mm × 30 mm electrostatic patches (Yunnan Medical Research Medical Biotechnology Co., Ltd., Yunnan, China) through the entire sleep period, one on each temple for 6 weeks. The voltage of the electrostatic patches dropped to half level (i.e., from initial 1,500 to 750 V) during the first half hour of use and thereafter maintained for 12 h (Wang et al., 2015). The electrostatic patch is a class II medical device approved by the State Food and Drug Administration (20160009). The electrostatic patches are mainly made of electret, which are polymeric discs that carry semi-permanent electrostatic charge and provide electrostatic potentials (Murthy et al., 2008).

All patients received three comprehensive assessments i.e., physical examination, nighttime polysomnographic sleep, daytime symptoms pre-treatment, and at 3 and 6 weeks after treatment initiation. Figure 1 depicts the procedures of this study.

### 2.3. Measures

#### 2.3.1. Clinical history and physical examination

Each patient completed a medical history and physical examination using a semi-structured questionnaire and a battery of clinical tests during their first visit to the sleep laboratory. Anthropometric measures included height and weight measurements according to standard procedures. Body mass index (BMI) was calculated based on weight (kg)/height (m^2^).

#### 2.3.2. Nighttime sleep

##### 2.3.2.1. Polysomnography

All patients included underwent two consecutive nights of PSG recording in sleep laboratory pre-treatment, and at 3 and 6 weeks post treatment. The sleep records were subsequently scored according to the international criteria of the American Academy of Sleep Medicine (Berry et al., 2020), by a technician blind to the subject’s characteristics and treatment status. For statistical analyses, we used the mean values of the sleep parameters obtained over the two consecutive nights.

##### 2.3.2.2. Insomnia severity

The Insomnia Severity Index (ISI) was used for assessing global self-reported insomnia severity. Higher total scores of ISI indicate more severe self-reported insomnia (Morin et al., 2011). The total scores of ISI ≤ 7 was defined as no insomnia, 8 ≤ ISI ≤ 14 was defined as subclinical insomnia, 15 ≤ ISI ≤ 21 was defined as moderate insomnia and 22 ≤ ISI ≤ 28 was defined as severity insomnia.

#### 2.3.3. Daytime symptoms

##### 2.3.3.1. Daytime sleepiness and fatigue

The multiple sleep latency test (MSLT) was used to assess objective daytime sleepiness. All participants underwent MSLT after the second night PSG recording in the sleep laboratory. The MSLT included four 20 min naps scheduled at 9:00, 11:00, 13:00, and 15:00 (Mashaqi et al., 2021). The mean sleep latency of the four naps was used as the measure of objective daytime sleepiness. Lower mean MSLT values indicate more objective daytime sleepiness. Self-reported daytime sleepiness was measured by the Epworth Sleepiness Scale (ESS). Higher total ESS scores indicate more self-reported daytime sleepiness (Johns, 1991). The Flinders Fatigue Scale (FFS) was used for assessing fatigue level. Higher total FFS scores indicate greater fatigue (Cameron et al., 2017).

##### 2.3.3.2. Mood status

The Beck Anxiety Inventory (BAI) and Beck Depression Inventory (BDI) (Lovibond and Lovibond, 1995) were used to assess anxiety and depressive symptoms, respectively. The higher total score indicates more severe symptoms of anxiety and depression.

##### 2.3.3.3. Attention assessment

The attention network test (ANT) was administered by E-prime software (Fan et al., 2002) to assess attention level in the morning following the second night PSG recording in the sleep laboratory. The test consisted of one practice block (24 trials) with full-feedback and three experimental blocks (96 trials) with no feedback. A spatial cue is presented at each trial, followed by an array of five arrows presented at either the top or the bottom of the computer screen. Patients must indicate the direction of the central arrow in the array of five. The cue that precedes the arrows can be non-existent, a center cue, a double cue (one presented at each of the two possible target locations), or a spatial cue that deterministically indicates the upcoming target location. In each trial, a cue was presented for 100 ms after a random duration of 400–1,600 ms. After the offset of the cue by 400 ms, the target flankers emerged, and persisted until the participant responded or for 1,700 ms if no response was given. Each network is assessed *via* reaction times (RTs). All RTs with erroneous input of *RT* > 1,500 ms and *RT* < 200 ms were excluded from the analysis. The scores for the three attention networks were calculated by subtracting the RTs of different conditions: Alerting = RT no cue – RT double cue; Orienting = RT central cue – RT spatial cue; Executive control = RT incongruent – RT congruent (Fan et al., 2005). Higher values of alerting and orienting indicate better alerting and orienting attention performance, respectively, whereas lower values of executive control indicate better executive control attention performance.

### 2.4. Statistical analysis

Data are presented as the mean ± standard deviation (SD) for continuous variables and frequency and percent for categorical variables. The independent *t-test* and Mann–Whitney *U* test were used for normally and non-normally distributed continuous variables respectively, and the χ^2^ test was used for categorical variables to compare pre-treatment characteristics between groups. One-way repeated measures analysis of variance (ANOVA) with time (pre-treatment, 3 and 6 weeks post treatment) as the repeated measure was conducted to assess the changes of self-reported insomnia severity, objective nighttime sleep, objective/self-reported daytime sleepiness, fatigue, mood status, and attention after electrostatic therapy. In order to examine whether the effects of electrostatic therapy on insomnia differ in insomnia patients with objective short sleep duration (ISSD) and normal sleep duration (INSD), we divided the whole sample based on the rounded median (390 min) of TST. ISSD was defined based on TST < 390 min, while INSD was defined based on TST ≥ 390 min. Two-way repeated measures analysis of covaricance (ANCOVA) (time × group) controlling for age, gender and BMI was conducted to examine the effects of electrostatic therapy on nighttime sleep and daytime symptoms between groups. Changes in sleep from pre- to post-treatment for varying times were calculated as (post-treatment values minus pre-treatment values)/pre-treatment values × 100 (%). The comparisons of the changes of TST and SE from pre- to post-treatment between ISSD and INSD were conducted using ANCOVA after controlling for age, gender and BMI. In this study, η^2^p was used to interpret the effect size as small (0.01), medium (0.06), or large (0.14). For all analyses, significance was set at alpha = 0.05 (2-tailed). The G*Power 3.1.9.2 program was used for power calculation (Teodorovici, 2022). All analyses except for power calculation were conducted using SPSS 26.0 (IBM Corp., Armonk, NY, USA).

## 3. Results

A total of 30 chronic insomnia patients completed a 6 week course of electrostatic therapy. Among the 30 chronic insomnia patients, 15 patients (50%) were women, with a mean age of 39.57 ± 14.03 years and the mean BMI of 21.76 ± 3.19 kg/m^2^. Based on the ISI score, most patients included were categorized as having moderate insomnia at pre-treatment. During the 6 week electrostatic therapy period, no participants reported any discomfort or adverse reactions related to electrostatic therapy.

### 3.1. Effects of electrostatic therapy on global self-reported insomnia severity

As shown in Table 1, one-way repeated measures ANOVA showed that total ISI score (Time effect *F* = 27.736, *P* < 0.001, η^2^*p* = 0.498) changed significantly with time. Overall, the severity level of insomnia decreased from moderate insomnia (ISI 19.24 ± 4.46) to subclinical insomnia (ISI 13.52 ± 6.54) after 6 weeks of electrostatic therapy. Paired comparisons showed that the ISI score decreased by 3.69 ± 3.81 points after 3 weeks treatment (*P* < 0.001) and 5.72 ± 4.47 points after 6 weeks treatment (*P* < 0.001) compared to the pre-treatment score. Furthermore, the total ISI score decreased at 6 weeks after beginning of treatment compared to that at 3 weeks after beginning of treatment (*P* = 0.016).

**TABLE 1:** Self-reported insomnia severity and objective nighttime sleep characteristics before and after electrostatic therapy.

Item	Pre-treatment	Treatment-3 weeks	Treatment-6 weeks	Time effect P	η^2^p
ISI	19.24 ± 4.46*^,†^	15.55 ± 5.39^‡^	13.52 ± 6.54	**<0**.**001**	0.498
TST (min)	398.13 ± 54.53^†^	401.04 ± 47.35^‡^	419.23 ± 39.00	**0**.**029**	0.114
SE (%)	82.33 ± 10.38^†^	83.11 ± 9.55^‡^	86.42 ± 7.28	**0**.**040**	0.105
SOL (min)	20.17 ± 17.88	16.44 ± 13.65	21.13 ± 23.82	0.417	0.030
WASO (min)	57.74 ± 39.54^†^	60.56 ± 40.38^‡^	40.88 ± 27.47	**0**.**013**	0.139
N1 (%)	10.58 ± 4.36	10.93 ± 3.60	10.61 ± 4.70	0.839	0.006
N2 (%)	45.23 ± 7.96	45.31 ± 5.67	45.72 ± 7.55	0.893	0.004
N3 (%)	22.85 ± 5.95	22.39 ± 6.05	21.85 ± 6.54	0.357	0.071
R (%)	21.35 ± 5.46	21.37 ± 2.78	21.80 ± 4.69	0.860	0.005

Data are presented as means ± standard deviation, η^2^p, effect size of time effect.

*P-values < 0.05 when pre-treatment vs. treatment-3 weeks.

^†^*P*-values < 0.05 when pre-treatment vs. treatment-6 weeks.

^‡^*P*-values < 0.05 when treatment-3 weeks vs. treatment-6 weeks.

Values in bold indicate a *p*-value < 0.05.

ISI, Insomnia Severity Index; N1%, percentage of non rapid eye movement sleep stage 1; N2%, percentage of non rapid eye movement sleep stage 2; N3%, percentage of non rapid eye movement sleep stage 3; R%, percentage of rapid eye movement sleep stage; SE, sleep efficiency; SOL, sleep onset latency; TST, total sleep time; WASO, wake time after sleep onset.

### 3.2. Effects of electrostatic therapy on objective nighttime sleep

Regarding electrostatic therapy effects on PSG-measured parameters (Table 1), one-way repeated measures ANOVA showed that TST (Time effect *F* = 3.748, *P* = 0.029, η^2^*p* = 0.114), WASO (Time effect *F* = 4.674, *P* = 0.013, η^2^*p* = 0.139), and SE (Time effect *F* = 3.406, *P* = 0.040, η^2^*p* = 0.105) changed significantly across different time points. Paired comparisons showed that TST increased by 21.11 ± 47.05 min (*P* = 0.020), SE increased by 4.09 ± 8.00% (*P* = 0.009) and WASO decreased by 16.87 ± 34.42 min (*P* = 0.012) after 6 weeks of electrostatic therapy, compared to pre-treatment. Furthermore, TST (*P* = 0.022) and SE (*P* = 0.045) increased and WASO (*P* = 0.007) decreased after 6 weeks of electrostatic therapy, compared to those after 3 weeks of treatment. However, no significant changes in objective sleep were observed after 3 weeks of treatment compared to pre-treatment. Moreover, sleep onset latency (SOL), percentage of non rapid eye movement sleep stage 1 (N1%), percentage of non rapid eye movement sleep stage 2 (N2%), percentage of non rapid eye movement sleep stage 3 (N3%) or percentage of rapid eye movement sleep stage (R%) did not change significantly.

### 3.3. Effects of electrostatic therapy on daytime symptoms

#### 3.3.1. Objective and self-reported daytime sleepiness and fatigue

One-way repeated measures ANOVA showed that ESS score (Time effect *F* = 4.856, *P* = 0.011, η^2^*p* = 0.148) and FFS score (Time effect *F* = 4.935, *P* = 0.015, η^2^*p* = 0.268) changed across different time points (Table 2). Paired comparisons showed that the ESS score decreased by 1.69 ± 4.38 points after 3 weeks of treatment (*P* = 0.047) and 2.10 ± 3.96 points after 6 weeks of treatment (*P* = 0.008) compared pre-treatment. In addition, after 6 weeks of electrostatic therapy, the mean ESS score decreased from 7.93 at pre-treatment to 5.83, indicating a decrease from mild to no self-reported daytime sleepiness. Similar to ESS scores, FFS scores decreased by 2.52 ± 5.32 points after 3 weeks of treatment (*P* = 0.017) and 3.86 ± 6.52 points after 6 weeks of treatment (*P* = 0.003), compared to pre-treatment. However, the MSLT did not change significantly after electrostatic therapy either at 3 weeks or 6 weeks of treatment.

**TABLE 2:** Daytime sleepiness, fatigue, mood status, and attention levels before and after electrostatic therapy.

Item	Pre-treatment	Treatment-3 weeks	Treatment-6 weeks	Time effect P	η^2^p
**Daytime sleepiness, and fatigue**					
MSLT (min)	11.56 ± 4.25	11.49 ± 4.07	11.43 ± 4.75	0.985	0.001
ESS	7.93 ± 5.05*^,†^	6.24 ± 5.64	5.83 ± 4.93	**0.011**	0.148
FFS	17.03 ± 6.34*^,†^	14.52 ± 5.01	13.17 ± 6.15	**0.015**	0.268
**Mood status**					
BAI	7.31 ± 8.38^†^	5.45 ± 5.93	4.28 ± 6.04	**0.031**	0.226
BDI	10.52 ± 6.29	8.55 ± 6.99	8.28 ± 9.11	0.104	0.154
**Attention levels**					
Mean RT (ms)	673.46 ± 124.78	650.08 ± 127.44	650.78 ± 130.27	0.226	0.058
Alerting	57.18 ± 33.44^†^	65.84 ± 38.78	79.41 ± 44.86	**0.012**	0.163
Orienting	51.39 ± 33.15	46.56 ± 34.17	52.76 ± 29.79	0.562	0.023
Executive control	122.85 ± 56.69*^,†^	102.21 ± 33.26	102.80 ± 42.39	**0.004**	0.200

Data are presented as means ± standard deviation, η^2^p, effect size of time effect.

*P-values < 0.05 when pre-treatment vs. treatment-3 weeks.

^†^*P*-values < 0.05 when pre-treatment vs. treatment-6 weeks.

^‡^*P*-values < 0.05 when treatment-3 weeks vs. treatment-6 weeks.

Values in bold indicate a *P*-value < 0.05.

BAI, Beck Anxiety Inventory; BDI, Beck Depression Inventory; ESS, Epworth Sleepiness Scale; FFS, Flinders Fatigue Scale; MSLT, multiple sleep latency test; RT, reaction time.

#### 3.3.2. Mood status

One-way repeated measures ANOVA showed that the total BAI scores changed across the different time points (Time effect *F* = 3.953, *P* = 0.031, η^2^*p* = 0.226; Table 2). Paired comparison showed that the BAI score decreased by 1.86 ± 5.04 points after 3 weeks of treatment (*P* = 0.057) and decreased by 3.03 ± 5.82 points after 6 weeks of treatment (*P* = 0.009) compared to pre-treatment. However, the total BDI scores did not change significantly across the different time points.

#### 3.3.3. Attention levels

One-way repeated measures ANOVA showed that alerting network (Time effect *F* = 4.875, *P* = 0.012, η^2^*p* = 0.163) and executive control network (Time effect *F* = 6.261, *P* = 0.004, η^2^*p* = 0.200) changed significantly across different time points (Table 2). Paired comparison showed that alerting network increased significantly after 6 weeks of electrostatic therapy (*P* = 0.003), and executive control network decreased after three (*P* = 0.006) and 6 weeks of electrostatic therapy (*P* = 0.013) compared to pre-treatment. However, mean RT or orienting network did not change significantly after electrostatic therapy. Correlation analyses showed that no significant relationship between ANT performance and sleep at pre-treatment or changes from pre- to post-treatment period.

### 3.4. Effects of electrostatic therapy on ISSD and ISND

**FIGURE 2:**
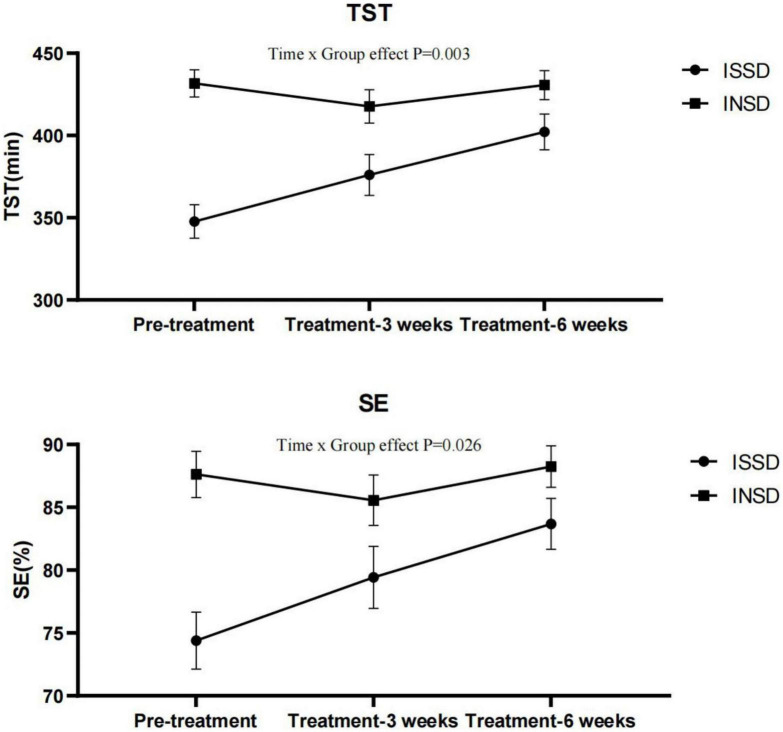
Total sleep time and sleep efficiency before and after electrostatic therapy in the ISSD and INSD groups. INSD, insomnia with normal sleep duration; ISSD, insomnia with short sleep duration; TST, total sleep time; SE, sleep efficiency. All data were presented after control for age, gender and BMI. Error bars present standard error.

**FIGURE 3:**
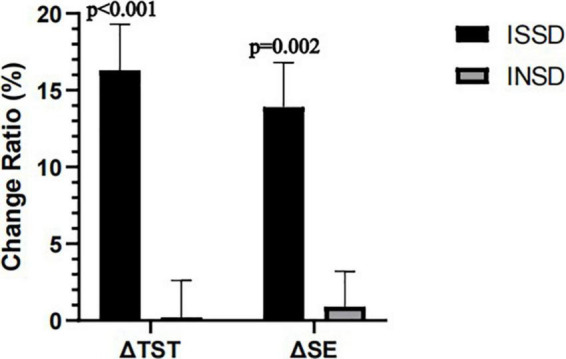
Changes in TST and SE from pre-treatment to 6 week post-treatment between ISSD and INSD groups. INSD, insomnia with objective normal sleep duration; ISSD, insomnia with objective short sleep duration; SE, sleep efficiency; TST, total sleep time. All data were presented after control for age, gender and BMI. Error bars present standard error.

As we found electrostatic therapy improved objective TST in patients with insomnia, we conducted a secondary analysis to examine the effects of electrostatic therapy on ISSD. TST (Time × Group effect *F* = 6.612, *P* = 0.003, η^2^*p* = 0.209) and SE (Time × Group effect *F* = 3.911, *P* = 0.026, η^2^*p* = 0.135) showed significant time × group interactions after controlling for age, gender, BMI, suggesting effects of electrostatic therapy on TST and SE differ in ISSD and INSD patients (Figure 2). In the ISSD group, TST increased by 28.33 ± 45.48 min after 3 weeks (*P* = 0.041) and 54.42 ± 40.89 min after 6 weeks of treatment (*P* < 0.001), compared to pre-treatment. Similar to TST, SE increased by 9.29 ± 7.15% after 6 weeks of treatment (*P* < 0.001). Furthermore, a significant increase in TST (*P* = 0.035) after 6 weeks of treatment compared to 3 weeks of treatment was also observed. In the INSD group, no significant changes in TST or SE were observed after 3 or 6 weeks of treatment, compared to pre-treatment. Figure 3 depicts the comparisons of changes in TST and SE from pre-treatment to 6 weeks of treatment between the ISSD and INSD groups. Compared to the INSD group, the changes in TST (16.30 ± 10.40% vs. 0.20 ± 10.2%, *p* < 0.001) and SE (13.90 ± 10.00% vs. 0.90 ± 9.80%, *p* = 0.002) from before to after 6 weeks of treatment were greater in the ISSD group after 6 weeks of electrostatic therapy. However, no significant time × group interactions were observed in terms of ISI score, other objective sleep parameters (i.e., WASO, SOL, N1%, N2%, N3%, and R%) and daytime symptoms (i.e., MSLT, ESS score, FFS score, BAI score, BDI score, and attention) after controlling for age, gender, and BMI (Supplementary Table 2). Supplementary Table 1 presents the demographic and clinical characteristics of the ISSD and INSD groups at baseline.

## 4. Discussion

To our knowledge, this is the first study to evaluate the treatment effects of electrostatic therapy on objective measured sleep and the most common daytime symptoms (i.e., fatigue, daytime sleepiness, mood status, and attention) in patients with chronic insomnia. Our preliminary findings suggest that electrostatic therapy improves both nighttime and daytime symptoms as well as objective sleep in patients with chronic insomnia; the nighttime sleep effect appears to be more robust in patients with objective short sleep duration.

Recently, a randomized double blind controlled trial investigated the effect of tACS on chronic insomnia. In this study, the authors found that the tACS intervention group had significantly higher response rate regarding the improvement of self-reported insomnia severity compared to the sham group. However, three participants had epileptiform discharges after tACS intervention (Wang et al., 2020). In the current study, our findings showed that self-reported insomnia severity rating by ISI improved from moderate to subclinical insomnia after 6 weeks of electrostatic therapy and with no significant side effects. Furthermore, given that objective sleep improved after 6 weeks but not 3 weeks of electrostatic therapy and ISI scores improved more in 6 weeks than 3 weeks of treatment, it is possible that the modest effect on the global insomnia severity might be associated with the relative short treatment period. Moreover, another explanation for the modest effect of electrostatic therapy on insomnia might be due to placebo effects. However, the significant improvement of objective sleep after electrostatic therapy based on two consecutive nights’ PSG recording argues against placebo effects. Future studies with inactive or placebo control group and longer treatment period are needed.

We found that objectively measured TST, SE, and WASO improved after 6 weeks of electrostatic therapy, which is consistent with a previous study that used tDCS to treat patients with depression and insomnia (Zhou et al., 2020). Furthermore, an early study with 10 insomnia patients showed significant reduction of sleep latency, as measured with electroencephalography after a 24 day (15 min/day) electrosleep treatment (Weiss, 1973). The inconsistency between studies could be due to different methodology, i.e., setting of electrical stimulation, participants included etc. In our study, we found that electrostatic therapy improved TST, SE, and WASO but not SOL, suggesting electrostatic therapy might be more efficacious for those patients with difficulty in maintaining sleep than in those with a primary complaint of difficulty falling asleep. Moreover, we found that objective sleep improved after 6 weeks but not 3 weeks of electrostatic therapy, suggesting short-term use of electrostatic therapy is not effective in insomnia.

Given the absence of a placebo control group, we cannot rule out that the improvement of objective sleep in patients with insomnia after electrostatic therapy was due to the placebo effect. Previous findings of the placebo effect on objective sleep in patients with insomnia were inconsistent (Mccall et al., 2003; Winkler and Rief, 2015; Yeung et al., 2018). The underlying mechanisms for the placebo effect on sleep was not clear. Regression to the mean, expectancy, the Hawthorne effect and physiologic changes produced by placebos might contribute to the placebo response (Mccall et al., 2005, 2011; Perlis et al., 2005). The placebo effect should be more robust in the early stage of intervention. However, the findings of our study that based on two consecutive nights PSG recording showed that the effect of electrostatic therapy on objective sleep was more robust in the later stage of intervention, thus it appears that the improvement of objective sleep after electrostatic therapy was not attribute to the placebo effect. Future rigorous randomized double-blind studies are needed to determine the effects of electrostatic therapy on sleep in patients with chronic insomnia.

In previous literature, Vgontzas et al. (2013) have proposed that insomnia with objective short sleep duration is a more severe biological phenotype of the disorder characterized by activation of the hypothalamic-pituitary-adrenal axis (HPA) and significant cardiometabolic morbidity and mortality. Interestingly, our findings showed that insomnia patients with objective short sleep duration had a better response to electrostatic therapy regarding objective nighttime sleep compared to insomnia with normal sleep duration. The findings suggest that electrostatic therapy might be a therapeutic choice for insomnia patients with objective short sleep duration who tend to respond less well to “first line” treatment of CBT-I (Bathgate et al., 2017; Vgontzas et al., 2020). The underlying mechanisms for better response to electrostatic therapy in this insomnia phenotype are not evident from this study. Future studies should explore possible mechanisms, for example effect of electrostatic therapy on the levels of cortisol (Vargas et al., 2018; Basta et al., 2022) that may explain the superior electrostatic therapy response in patient with ISSD. However, there was no difference regarding the improvement of self-reported insomnia severity measured by ISI between these two groups. The reasons for the discrepancy between effects on objective and self-reported sleep are not clear, but previous studies have suggested that self-reported severity of insomnia does not associate with objective sleep duration in patients with insomnia (Sun et al., 2022).

Fatigue, daytime sleepiness and attention impairment are the most common daytime symptoms in patients with insomnia. In our study, we found that the total scores of FFS and ESS decreased significantly after 3 and 6 weeks of electrostatic therapy, and attention improved significantly after 6 weeks of electrostatic therapy, suggesting electrostatic therapy improves daytime symptoms (i.e., fatigue, self-reported daytime sleepiness, and attention) in chronic insomnia patients. Furthermore, the findings of decreased levels of self-reported daytime sleepiness and fatigue after electrostatic therapy suggests that electrostatic therapy is not associated with hangover and daytime sleepiness common side effects of medications used to treat insomnia. Moreover, though attention as measured by ANT improved significantly, we found there was no significant association between ANT and insomnia. The non-significant association between ANT and sleep among insomnia patients were consistent with previous studies (Perrier et al., 2015). It appears that ANT is a measure for assessing attention levels but might not be a reliable measure for assessing neural mechanisms in patients with insomnia. Future studies using neuroimaging measures are needed to examine the underlying neural mechanisms of nighttime sleep improvement in patients with insomnia. In addition, our findings suggest that electrostatic therapy improves anxiety level, which is consistent with previous studies (Shekelle et al., 2018; Kim et al., 2021). However, we did not observe electrostatic therapy improve depressive symptoms, this could be associated with the relative low level of depressive symptoms at pre-treatment.

The mechanisms for electrostatic therapy on insomnia are not well-understood. It has been reported that electrical stimulation by affecting the central (i.e., increased cerebral blood flow in the locus coeruleus and thalamus, broadly modulates functional connectivity in the brain’s default mode network) (Feusner et al., 2012; Gense de Beaufort et al., 2012) and peripheral nervous system (increases relative parasympathetic-sympathetic drive) (Asamoah et al., 2019), as well as neurotransmitter and hormonal levels [i.e., decreased cortisol levels, increased serotonin release levels, modulation of norepinephrine release and increased gamma-aminobutyric acid (GABA) release] (Krupitsky et al., 1991; Liss and Liss, 1996; Cho et al., 2016; Roh and So, 2017; Wagenseil et al., 2018) may improve sleep and mood. Future studies with rigorous design should be conducted to examine the mechanisms of electrostatic therapy on improving insomnia.

## 5. Strengths and limitations

There are several strengths and limitations of the current study that merit discussion. Strengths include the careful selection of patients, a rigorous experimental protocol including two, 8 h consecutive nights of in-lab PSG and insomnia-related daytime function assessments. Several limitations also need to be acknowledged. First, the open-label design and the lack of an inactive or placebo control group precludes any definitive inference on the magnitude and direction of the therapeutic effects observed. However, the significant improvement of objective sleep based on two consecutive nights’ PSG recording after electrostatic therapy may eliminate the concerns of the placebo effects of electrostatic therapy. In our previous studies with three consecutive nights and two single nights separated by several years suggest that sleep duration variables, particularly total sleep time are stable in patients with insomnia (Gaines et al., 2015). Thus, we believe that the improvement of objective sleep based on two consecutive nights’ PSG recording is due to the treatment effects of electrostatic therapy but not the spontaneous resolution or placebo effects over time. Second, we did not examine the long-term effects of electrostatic therapy for chronic insomnia. Third, most patients included were mild-to-moderate insomnia patients, our findings cannot be generalized to patients with severe insomnia. Fourth, we did not collect the information of participants’ life events, which may affect sleep individually. Future prospective randomized controlled studies that include a full spectrum of insomnia patients with a long-term follow-up and with the consideration of life events are needed to examine the effect of electrostatic therapy on insomnia real or placebo effect and the electrostatic therapy effects on objective sleep and insomnia daytime symptoms in patients with chronic insomnia.

## 6. Conclusion

Our findings suggest that electrostatic therapy improves both nighttime sleep and daytime symptoms in patients with chronic insomnia. Electrostatic therapy might be a therapeutic choice for insomnia patients with difficulty in maintaining sleep, especially in those with objective short sleep duration who tend to respond less well to “first line” treatment of CBT-I.

## Data availability statement

The original contributions presented in this study are included in the article/Supplementary material, further inquiries can be directed to the corresponding author.

## Ethics statement

The studies involving human participants were reviewed and approved by the Institutional Review Board of Shantou University Mental Health Center. The patients/participants provided their written informed consent to participate in this study.

## Author contributions

YD: formal analysis, data curation, writing – original draft, and visualization. QQ, AV, and MB: writing – review and editing. LC and QS: investigation and acquisition of data. BC: project administration. YL: conceptualization, methodology, supervision, and writing – review and editing. All authors contributed to the article and approved the submitted version.
