# Supplementary material for: Effects of electrostatic therapy on nighttime sleep and daytime symptoms in patients with chronic insomnia: Evidences from an open label study
Source: Front Neurosci. 2023 Jan 6;16:1047240. doi: 10.3389/fnins.2022.1047240 (PMC9853294; doi:10.3389/fnins.2022.1047240)
Supplement: Supplementary file 1 [file Data_Sheet_1.docx]

**Supplementary Table 1. Baseline demographic and clinical characteristics between the ISSD and INSD groups**

| Item | All (N=30) | ISSD  (N=12) | INSD  (N=18) | P |
| --- | --- | --- | --- | --- |
| Age, years | 39.57 ± 14.03 | 42.50 ± 10.60 | 37.61 ± 15.90 | 0.359 |
| Gender (female, %) | 15 (50) | 6 (50) | 9 (50) | 1.000 |
| BMI, kg/m2 | 21.76 ± 3.19 | 22.06 ± 2.67 | 21.56 ± 3.55 | 0.679 |
| Baseline SBP, mmHg | 114.72 ± 11.82 | 116.18 ± 12.90 | 113.57 ± 11.26 | 0.595 |
| Baseline DBP, mmHg | 75.68 ± 8.30 | 77.00 ± 9.33 | 74.64 ± 7.58 | 0.492 |
| ISI score | 19.24 ± 4.46 | 19.50 ± 4.54 | 19.06 ± 4.40 | 0.791 |
| ESS score | 7.93 ± 5.05 | 6.75 ± 5.56 | 8.39 ± 4.78 | 0.396 |
| FFS score | 17.03 ± 6.34 | 15.67 ± 7.56 | 17.61 ± 5.45 | 0.419 |
| BAI score | 7.31 ± 8.38 | 6.50 ± 5.16 | 7.72 ± 9.90 | 0.692 |
| BDI score | 10.52 ± 6.29 | 10.17 ± 6.48 | 10.78 ± 6.16 | 0.796 |

Note: data are presented as means ± standard deviation, or sample size (percentage).

Abbreviations: BAI=Beck Anxiety Inventory; BDI=Beck Depression Inventory; BMI=Body mass index; DBP=Diastolic blood pressure; ESS=Epworth Sleepiness Scale; FFS=Flinders Fatigue Scale; INSD=Insomnia with objective normal sleep duration; ISI=Insomnia Severity Index; ISSD=Insomnia with objective short sleep duration; SBP =Systolic blood pressure

**Supplementary Table 2.** **Nighttime sleep, daytime sleepiness, fatigue, mood status and attention levels before and after electrostatic therapy between ISSD and INSD groups**

|  | ISSD (N=12) | | | INSD (N=18) | | |  |  |  |  |  |  |
| --- | --- | --- | --- | --- | --- | --- | --- | --- | --- | --- | --- | --- |
|  | Pre-treatment | 3 weeks after treatment | 6 weeks after treatment | Pre-treatment | 3 weeks after treatment | 6 weeks after treatment | Group effect P | η²p1 | Time effect P | η²p2 | Time×Group effect P | η²p3 |
| Nighttime sleep | | | | | | | | | | | | |
| ISI | 19.55 ± 4.76^*, †^ | 15.64 ± 5.87 | 14.82 ± 6.05 | 19.06 ± 4.40^*, †^ | 15.50 ± 5.25^‡^ | 12.72 ± 6.88 | 0.781 | 0.003 | 0.204 | 0.064 | 0.407 | 0.037 |
| TST, min | 347.63 ± 40.43^*, †^ | 372.79 ± 52.10^‡^ | 402.27 ± 46.79 | 431.79 ± 31.85 | 419.88 ± 33.60 | 430.54 ± 28.97 | **<0.001** | 0.450 | 0.117 | 0.082 | **0.003** | 0.209 |
| SE, % | 74.05 ± 10.79^†^ | 78.44 ± 10.46 | 83.60 ± 9.20 | 87.84 ± 5.29 | 86.22 ± 7.69 | 88.29 ± 5.14 | **0.002** | 0.334 | 0.101 | 0.088 | **0.026** | 0.135 |
| SOL, min | 26.23 ± 24.18 | 15.52 ± 14.31 | 21.94 ± 29.51 | 16.13 ± 11.13 | 17.06 ± 13.57 | 20.58 ± 20.10 | 0.627 | 0.010 | 0.868 | 0.006 | 0.271 | 0.051 |
| WASO, min | 84.48 ± 42.02^†^ | 82.19 ± 42.57^‡^ | 51.02 ± 30.09 | 39.92 ± 26.14 | 46.14 ± 32.54 | 34.11 ± 24.08 | **0.002** | 0.329 | 0.186 | 0.065 | 0.223 | 0.058 |
| N1, % | 12.34 ± 4.95 | 12.39 ± 2.83 | 10.70 ± 3.43 | 9.40 ± 3.59 | 9.95 ± 3.80 | 10.55 ± 5.48 | 0.221 | 0.059 | 0.836 | 0.007 | 0.081 | 0.096 |
| N2, % | 44.86 ± 9.01 | 44.93 ± 5.81 | 46.64 ± 8.40 | 45.47 ± 7.44 | 45.57 ± 5.73 | 45.11 ± 7.11 | 0.975 | <0.001 | 0.884 | 0.005 | 0.545 | 0.024 |
| N3, % | 22.11 ± 5.15 | 21.51 ± 5.86 | 20.47 ± 5.46 | 23.34 ± 6.52 | 22.98 ± 6.27 | 22.77 ± 7.18 | 0.567 | 0.013 | 0.973 | 0.001 | 0.839 | 0.007 |
| R, % | 20.68 ± 5.22 | 21.18 ± 2.41 | 22.19 ± 5.15 | 21.80 ± 5.72 | 21.50 ± 3.05 | 21.54 ± 4.49 | 0.944 | <0.001 | 0.633 | 0.018 | 0.773 | 0.010 |
| Daytime sleepiness and fatigue | | | | | | | | | | | | |
| MSLT, min | 10.79 ± 4.09 | 10.80 ± 4.17 | 12.38 ± 4.17 | 12.10 ± 4.40 | 11.98 ± 4.05 | 10.76 ± 5.13 | 0.688 | 0.007 | 0.262 | 0.054 | 0.054 | 0.114 |
| ESS | 7.18 ± 5.62 | 4.82 ± 5.62 | 5.00 ± 5.22 | 8.39 ± 4.78 | 7.11 ± 5.62 | 6.33 ± 4.83 | 0.629 | 0.010 | 0.306 | 0.048 | 0.708 | 0.014 |
| FFS | 16.09 ± 7.78 | 15.36 ± 6.22 | 14.55 ± 7.94 | 17.61 ± 5.45 | 14.00 ± 4.23 | 12.33 ± 4.83 | 0.433 | 0.026 | 0.465 | 0.031 | 0.265 | 0.054 |
| Mood status | | | | | | | | | | | | |
| BAI | 6.64 ± 5.39 | 5.27 ± 4.80 | 3.45 ± 3.27 | 7.72 ± 9.90 | 5.56 ± 6.66 | 4.78 ± 7.28 | 0.812 | 0.002 | 0.096 | 0.184 | 0.881 | 0.011 |
| BDI | 10.09 ± 6.79 | 9.00 ± 7.03 | 9.00 ± 9.74 | 10.78 ± 6.16 | 8.28 ± 7.15 | 7.83 ± 8.97 | 0.704 | 0.006 | 0.564 | 0.049 | 0.805 | 0.019 |
| Attention levels | | | | | | | | | | | | |
| Mean RT, ms | 673.41 ± 116.45 | 622.71 ± 119.87 | 648.59 ± 140.35 | 673.49 ± 133.47 | 667.18 ± 132.80 | 652.14 ± 128.29 | 0.361 | 0.040 | 0.706 | 0.016 | 0.437 | 0.039 |
| Alerting | 46.29 ± 33.18 | 49.82 ± 40.53 | 75.88 ± 40.24 | 63.98 ± 32.79 | 75.86 ± 35.24 | 81.62 ± 48.67 | 0.208 | 0.074 | 0.287 | 0.058 | 0.623 | 0.022 |
| Orienting | 44.35 ± 39.91 | 28.07 ± 36.09 | 46.35 ± 39.15 | 55.79 ± 28.67 | 58.12 ± 28.19 | 56.77 ± 22.69 | 0.231 | 0.068 | 0.073 | 0.117 | 0.192 | 0.075 |
| Executive control | 113.41 ± 32.01 | 101.17 ± 25.60 | 95.94 ± 36.89 | 128.75 ± 68.16^*^ | 102.86 ± 38.07 | 107.09 ± 46.13 | 0.295 | 0.052 | 0.480 | 0.034 | 0.436 | 0.039 |

Note: Data are presented as means ± standard deviation. Above P valve were presented after adjusting for age, gender, BMI. η²p1, effect size of group effect. η²p2, effect size of time effect. η²p3, effect size of time×group effect. * indicated p values <0.05 when pre-treatment vs. treatment-3 weeks. † indicated p values <0.05 when pre-treatment vs. treatment-6 weeks. ‡ indicated p values <0.05 when treatment-3 weeks vs treatment-6 weeks. Values in bold indicate a p-value < 0.05.

Abbreviations: BAI=Beck Anxiety Inventory; BDI=Beck Depression Inventory; ESS=Epworth Sleepiness Scale; FFS=Flinders Fatigue Scale; INSD=Insomnia with objective normal sleep duration; ISI=Insomnia Severity Index; ISI=Insomnia Severity Index; ISSD=Insomnia with objective short sleep duration; N1%=Percentage of non rapid eye movement sleep stage 1; N2%=Percentage of non rapid eye movement sleep stage 2; N3%=Percentage of non rapid eye movement sleep stage 3; R%=Percentage of rapid eye movement sleep stage; RT=Reaction time; SE=Sleep efficiency; SOL=Sleep onset latency; TST=Total sleep time; WASO=Wake time after sleep onset
